# Revana: a comprehensive tool for regulatory variant analysis and visualization of cancer genomes

**DOI:** 10.1093/bioinformatics/btac831

**Published:** 2022-12-28

**Authors:** Elias Ulrich, Stefan M Pfister, Natalie Jäger

**Affiliations:** Division of Pediatric Neurooncology, Hopp Children’s Cancer Center Heidelberg (KiTZ), German Cancer Research Center (DKFZ), Heidelberg 69120, Germany; German Cancer Consortium (DKTK), German Cancer Research Center (DKFZ), Heidelberg 69120, Germany; Division of Pediatric Neurooncology, Hopp Children’s Cancer Center Heidelberg (KiTZ), German Cancer Research Center (DKFZ), Heidelberg 69120, Germany; German Cancer Consortium (DKTK), German Cancer Research Center (DKFZ), Heidelberg 69120, Germany; Department of Pediatric Oncology, Hematology and Immunology, Heidelberg University Hospital, Heidelberg 69120, Germany; Division of Pediatric Neurooncology, Hopp Children’s Cancer Center Heidelberg (KiTZ), German Cancer Research Center (DKFZ), Heidelberg 69120, Germany; German Cancer Consortium (DKTK), German Cancer Research Center (DKFZ), Heidelberg 69120, Germany

## Abstract

**Motivation:**

As non-coding driver mutations move more into the focus of cancer research, a comprehensive and easy-to-use software solution for regulatory variant analysis and data visualization is highly relevant. The interpretation of regulatory variants in large tumor genome cohorts requires specialized analysis and visualization of multiple layers of data, including for example breakpoints of structural variants, enhancer elements and additional available gene locus annotation, in the context of changes in gene expression.

**Results:**

We introduce a user-friendly tool, Revana (REgulatory Variant ANAlysis), that can aggregate and visually represent regulatory variants from cancer genomes in a gene-centric manner. It requires whole-genome and RNA sequencing data of a cohort of tumor samples and creates interactive HTML reports summarizing the most important regulatory events.

**Availability and implementation:**

Revana is implemented in R and JavaScript. It is available for download as an R package under <https://github.com/KiTZ-Heidelberg/revana>. Sample results can be viewed under <https://github.com/KiTZ-Heidelberg/revana-demo-report> and a short walkthrough is available under <https://github.com/KiTZ-Heidelberg/revana-demo-data>.

**Supplementary information:**

Supplementary data are available at *Bioinformatics* online.

## 1 Introduction

The sensitive and specific detection of driver mutations is an important task in cancer genomics. While variants in the protein-coding space are nowadays mostly relatively easy to detect, the reliable detection of non-coding driver variants is more challenging yet equally relevant (Rheinbay *et al.*, 2020). Non-coding drivers involve diverse mechanisms and usually function by altering the expression of certain, often spatially proximal genes. They do not alter the amino-acid structure of the translated proteins and are thus called regulatory variants. Prominent examples are recurrent single-nucleotide variants (SNVs) in the promoter of *TERT* (Horn *et al.*, 2013; Huang *et al.*, 2013), that upregulate the gene and thus maintain telomeres across many cancer entities (Remke, 2013; Vinagre *et al.*, 2013).

Another mechanism is enhancer hijacking, where structural variants (SVs) alter the surrounding 3D DNA structure of genes and juxtapose them to highly active regulatory regions like gene enhancers. By upregulating *GFI1/GFI1B* and *PRDM*, enhancer hijacking drives pediatric medulloblastoma (Northcott *et al.*, 2014, 2017) among many other tumor entities (Haller *et al.*, 2019; Helmsauer *et al.*, 2020; Montefiori *et al.*, 2021).

Previously developed methods to discover regulatory variants from whole-genome (WGS) and RNA sequencing (RNA-seq) data include cis-X (Liu *et al.*, 2020). It relies on allele specific as well as outlier high expression (OHE) to identify genes activated in cis, and subsequently associates them with potential driver mutation candidates. In contrast, CESAM (Weischenfeldt *et al.*, 2017) uses linear regression of gene expression on somatic copy number alterations (CNAs), while Rheinbay *et al.* (2020) introduced the concepts of recurrent SV breakpoints and SV juxtaposition.

We provide an R package for the visualization and analysis of regulatory variants in cancer genome cohorts. Besides combining several features of the existing methods, we added consideration of gene-specific regulatory regions, sample aggregation and comprehensive result evaluation features, that no existing solution provides yet to the best of our knowledge. Subsequent analysis of a well-described, published cohort of medulloblastoma genomes demonstrates that Revana (REgulatory Variant ANAlysis) can effectively detect and illustrate known regulatory variants.

## 2 Implementation

### 2.1 Data input and preparation

Revana requires processed data from deep WGS and RNA-seq for a cohort of tumor samples as input. Tab-separated value files providing SNP markers, copy numbers, structural variants, somatic SNVs and InDels and gene expression feature counts are used as input (hg19 or hg38) and need to be created prior to the application of Revana with any of the many available tools (see Supplementary Material).

### 2.2 Identification of cis-activated genes

Revana adopts the approach used by Liu *et al.*, combining allele-specific expression (ASE) and OHE to identify cis-activated genes that are potentially subject to variant-based upregulation. ASE statistics calculated from heterozygous SNP marker allele frequencies are compared to a balanced expression model established by Liu *et al.* OHE is determined using a leave-one-out test against a pre-filtered set of other tumor samples of the cohort and eliminates the need for a precalculated gene expression reference matrix.

### 2.3 Associating genes with non-coding, regulatory variants

To identify potential regulatory somatic variants responsible for the upregulation of cis-activated genes, Revana matches genes and variants using three alternative approaches. As also utilized in cis-X, it first considers variants of the same topologically associating domain (TAD) in which the gene is located. Cis-X prioritized SVs and CNAs and only considered somatic SNVs/InDels if no other variant type could be detected. Yet, since point mutations are frequent events in cancer genomes, their presence alone cannot determine genuine regulatory variant candidates. We found, that a more rigorous selection of SNVs by transcription factor binding site analysis with FIMO (Grant *et al.*, 2011) as used in cis-X, was not able to improve the predictive power with respect to cis-activation. Thus, we provide it only as a descriptive feature.

Conversely, Revana associates genes irrespective of their cis-activation status with all types of variants from the same TAD. Thus, the strength of association for each mutation type with gene cis-activation can be estimated and compared (e.g. odds ratio, OR_SNV/InDel_ = X, ORSV = Y).

A second, complementary approach exploits gene-specific regulatory regions, so-called GeneHancers (Fishilevich *et al.*, 2017). Promoter and enhancer elements of each gene under investigation allow for the matching of mutations beyond the TAD as well as a more selective choice of point mutations within the same TAD. Optionally, ChiP-seq data, ideally tissue-matched, can provide regulatory regions specific to the samples under investigation and is then used for gene-to-variant association, result annotation and visualization.

### 2.4 Automatic report generation and data visualization

Subsequently, Revana creates a comprehensive HTML report that helps evaluate, prioritize and visualize candidate events and allows to compare results of different tumor subgroups.

JavaScript-powered interactive illustrations show recurrently cis-activated genes across the cohort, highlight samples with different types of potential regulatory variants and provide multiple sorting and filtering options. This allows to prioritize between the discovered gene candidates and quickly elucidate genes repeatedly affected by comparable regulatory mechanisms. Moreover, recurrent SV juxtapositions can provide further support for systematically involved genetic mechanisms.

For each discovered gene candidate, a separate document shows the recurrence of mutations, the expression and allelic imbalance of the gene across the investigated samples and tumor subgroups as well as more explicit statistics. A gene locus plot illustrates all present somatic variants and thus helps interpreting the detected somatic genomic events.

Finally, the integrative genome viewer (IGV) plugin (Robinson *et al.*, 2022), facilitates interactive inspection of the gene TAD, other distant breakpoints and any available alignment directly within the browser.

## 3 Results

**Fig. 1.**
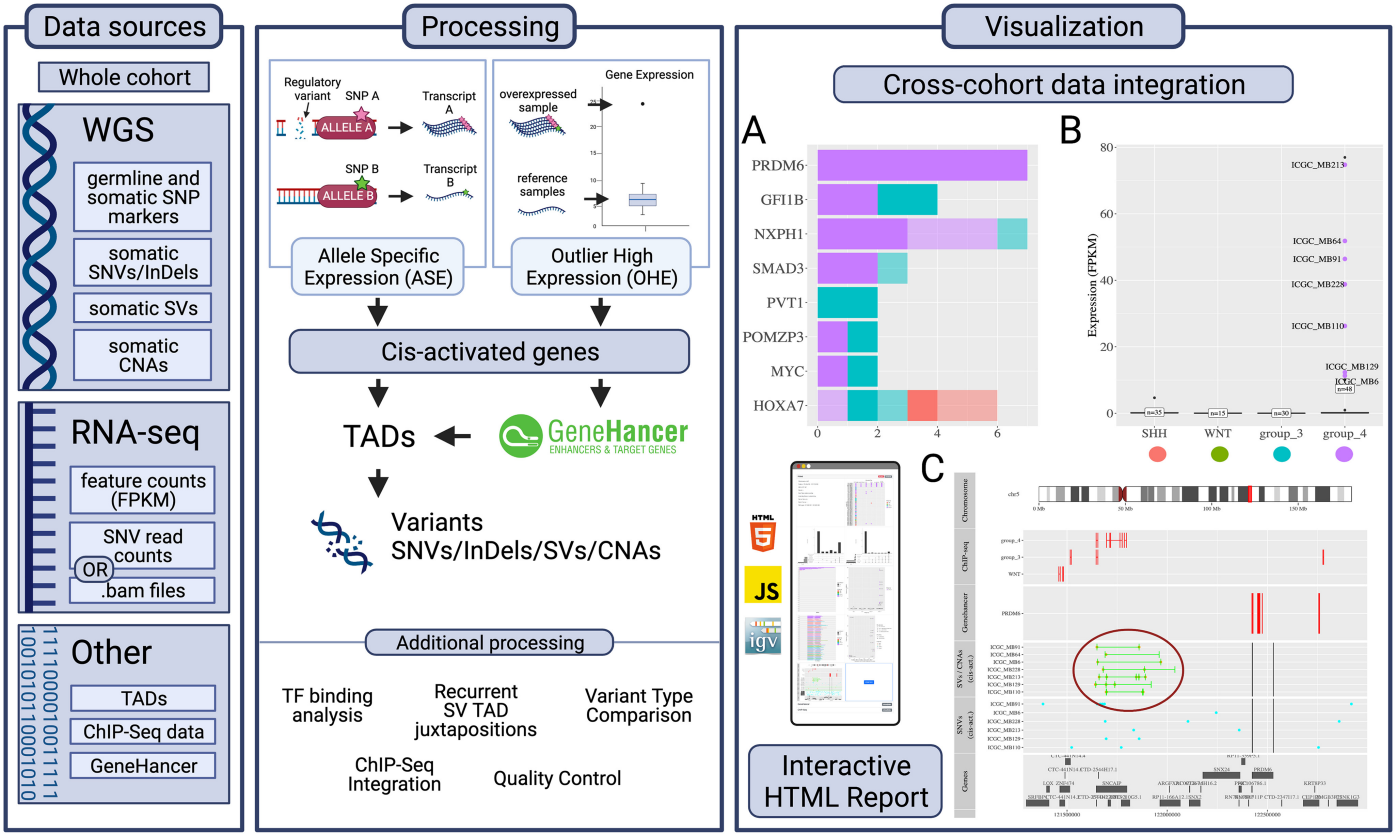
Workflow of Revana: (**A**) Cis-activated samples by gene: solid—cis-activated with GeneHancer-matched variant, transparent—cis-activated without GeneHancer-matched variant; (**B**) *PRDM6* expression across medulloblastoma subgroups; (**C**) *PRDM6* gene locus plot—recurrent *SNCAIP* tandem duplications cause upregulation

To demonstrate Revana’s capabilities, we applied it to our cohort of 128 medulloblastomas from the International Cancer Genome Consortium (ICGC PedBrain) and identified a total of 3952 cis-activated candidate genes across the cohort, of which 1502 carry potential regulatory variants. Revana’s filtering and sorting features quickly revealed the known top candidates (Fig. 1A). These include the two well-studied enhancer hijacking loci *PRDM6* (Fig. 1B and C) (6/7 cases recognized; one case lacked sufficient SNP markers for ASE detection) (Northcott *et al.*, 2017) and *GFI1B* (4/4 cases) (Northcott *et al.*, 2014) as well as characteristic *PVT1* fusions (Northcott *et al.*, 2012). Gene locus illustrations summarize these genomic events (Fig. 1C). Our results conclusively demonstrate that Revana can identify known and novel non-coding regulatory variants in cohorts of cancer genomes.

*Financial Support*: none declared.

*Conflict of Interest*: none declared.

## Supplementary Material

btac831_Supplementary_Materials

## Data Availability

The data underlying this article is available in the GitHub repositories at https://github.com/KiTZ-Heidelberg/revana-demo-data and https://github.com/KiTZ-Heidelberg/revana-demo-report.
