# Supplementary material for: Revana: a comprehensive tool for regulatory variant analysis and visualization of cancer genomes
Source: Bioinformatics. 2022 Dec 28;39(1):btac831. doi: 10.1093/bioinformatics/btac831 (PMC9822537; doi:10.1093/bioinformatics/btac831)
Supplement: btac831_Supplementary_Materials [file btac831_supplementary_materials.pdf]

Supplementary Material

Revana: comprehensive tool for regulatory variant analysis and visualization of cancer genomes

Elias Ulrich[1*], **Stefan M. Pfister**[1,2], **Natalie Jäger**[1]
[1]Hopp Children's Cancer Center Heidelberg (KiTZ) & Division of Pediatric Neurooncology, German Cancer Research Center (DKFZ), Heidelberg, Germany and German Cancer Consortium (DKTK), German Cancer Research Center (DKFZ), Heidelberg, Germany
[2]Department of Pediatric Oncology, Hematology and Immunology, Heidelberg University Hospital, Heidelberg, Germany
*To whom correspondence should be addressed.

Short description *(Github and Vignette only / not in supplementary)*

Revana is a comprehensive tool for regulatory variant detection in cancer genomes. It can detect, analyse, and illustrate regulatory, non-coding variants of different kinds. It works on whole genome (WGS) and RNA sequencing (RNA-Seq) data of a cohort of tumor samples and creates interactive HTML reports summarizing the most important regulatory events.

Installation

Revana can be installed from the R console with the following command:

```
if (!requireNamespace("remotes", quietly = TRUE))
    install.packages("remotes")
remotes::install_github("https://github.com/KiTZ-Heidelberg/revana")
```

For more details about the installation or any trouble shooting see the demonstration walkthrough under <https://github.com/KiTZ-Heidelberg/revana-demo-data>

Input formats

The following files are used as input for Revana. Some files are required once per included sample and others only once per cohort of tumor genomes.

Required files for each tumor sample

Marker file

The marker file contains all germline single nucleotide polymorphisms and all somatic single nucleotide variants of the sample. It also provides the reference and alternate read counts for WGS and RNA-seq. If the RNA-seq read counts are not readily available Revana can generate them from .bam files (see Create Required reference/input data with Revana). The file is formatted as tab separated values with a header and contains the following columns:

Column name	Description	Example
chrom	chromosome of the SNP in “UCSC style”	chr12
pos	genomic position of the SNP	12000678
ref	reference base at SNP position	A
alt	alternate base at SNP position	T
reads.WGS.ref	number of WGS reads with reference base	87
reads.WGS.alt	number of WGS reads with alternate base	35
reads.RNA.ref	number of RNA reads with reference base	127
reads.RNA.alt	number of RNA reads with alternate base	5

Copy number file

The copy number file contains somatic coverage ratios (as surrogate for copy numbers) and their respective genomic regions. The file is formatted as tab separated values with a header and contains the following columns:

**Column name**	**Description**	**Example**
chrom	chromosome of the genomic region in "UCSC style"	chr12
start	start of the genomic region	11000000
end	end of the genomic region	12000000
cov_ratio	coverage ratio of the genomic region	1.4

CNA file

The CNA file contains somatic copy number alterations (CNAs), that is regions with divergent ploidy or copy number in the somatic tissue. We have previously used regions with copy number alterations with $\Delta_{\text{copy number}} > 0.3$ but any threshold can be used. The file is formatted as tab separated values with a header and contains the following columns:

**Column name**	**Description**	**Example**
chrom	chromosome of the genomic region of the CNA in "UCSC style"	chr12
start	start of the genomic region of the CNA	11000000
end	end of the genomic region of the CNA	12000000
*copy_number**	*copy number of the genomic region of the CNA*	*2.8*
*CNA_type**	*Annotation of the CNA type from the CNA caller*	*hom_del*
*log2**	*log2 of the coverage ratio of the genomic region of the CNA*	*0.48*

* These columns are annotative only. Thus, no calculations are based on their values.

<u>Difference between the Copy number file and the CNA file:</u>

The **copy number** file provides Revana with the determined read coverage ratio of genomic ranges across the entire genome. The **CNA file** provides only those genomic regions that are considered as abberant by the user. Therefore, the filtering of this input files determines which copy number alterations are considered as tumor-specific somatic genomic events.

Somatic SNVs file

This file contains somatic single nucleotide variants (SNVs) and small insertions and deletions (InDels). The file is formatted as tab separated values with a header and contains the following columns:

**Column name**	**Description**	**Example**
chrom	chromosome of the SNV or Indel in "UCSC style"	chr12
pos	genomic position of the SNV or Indel	12000678
ref	reference base at SNV position	A
alt	alternate base at SNV position	T
*CNA_type**	*Annotation of the CNA type from the CNA caller*	*hom_del*
*log2**	*log2 of the coverage ratio of the genomic region of the CNA*	*0.48*

Insertions and deletions (InDels) should be listed in the following format:

**Variant**	**Ref Value**	**Alt Value**
Small insertion of GT	A	AGT
Small deletion of ATG	CATG	C

Expression file

This file contains RNA-seq expression feature counts measured in fragments per kilobase per one million reads (FPKM). The included gene names have to be in accordance with the used gene annotation (e.g. Gencode). The file is formatted as tab separated values with a header and contains the following columns:

**Column name**	**Description**	**Example**
gene_name	Name of the gene	PRDM6
FPKM	Gene expression in FPKM	410.2

SV file

This file contains somatic structural variants (SVs) and their respective genomic breakpoints. The file is formatted as tab separated values with a header and contains the following columns:

Column name	Description	Example
chrom1	chromosome of the first breakpoint of the SV in “UCSC style”	chr12
pos1	genomic position of the first breakpoint of the SV	43100001
chrom2	chromosome of the second breakpoint of the SV in “UCSC style”	chr12
pos2	genomic position of the second breakpoint of the SV	60500432
*sv_type**	*Annotation of the SV type from the SV caller*	*INV*
*eventInversion**	*Annotation if the strands affected by the SV event were inverted*	*INV*

* These columns are annotative only. Thus, no calculations are based on their values.

Required input files for the whole cohort

Paths file

This file includes the paths of the above-described input files and the ID of the respective sample. The file is formatted as tab separated values with a header and contains the following columns:

Column name	Description	Example
sample_id	ID of the tumor sample	ICGC_MB1
CNA_file	Path to the CNA file	/path/to/CNA_file.txt
somatic_SNV_file	Path to the somatic SNV file	/path/to/somatic_SNV_file.txt
expression_file	Path to the expression file	/path/to/expression_file.txt
SV_file	Path to the SV file	/path/to/SV_file.txt
copy_number_file	Path to the copy number file	/path/to/copy_number_file.txt

TAD file

This file contains the genomic regions of topologically associated domains (TADs) for the tissue type under investigation. The file is formatted as tab separated values with header and contains the following columns:

Column name	Description	Example
chrom	chromosome of TAD region in “UCSC style”	chr12
start	start of TAD region	12345678
end	end of TAD region	12387654

ChIP-seq file

This file contains regulatory active regions identified via chromatin immune precipitation (ChIP-Seq). Any other input that contains regulatory regions is fine and can be used alternatively. The file is formatted as tab separated values with header and contains the following columns:

Column name	Description	Example
chrom	chromosome of regulatory region in “UCSC style”	chr12
start	start of the regulatory region	34897132
end	end of the regulatory region	34899132
*cluster**	*annotation column*	*WNT*

* These columns are annotative only. Thus, no calculations are based on their values.

If no ChIP-seq data is available, set the *chipseq_file_path* argument in the *run_analysis* function to *NULL*.

FAQ: Do I need to provide all these files to use Revana?

Revana is designed to analyse regulatory variants based on whole-genome and RNA sequencing data. The detection of regulatory variants is generally a more sophisticated question within the genome analysis workflows of sequenced tumor samples. Therefore, in most cases, mutation data (from WGS) such as somatic SNVs/InDels, structural variants, copy number profiles as well as expression data (from RNA sequencing) will already be available. If not, there are many established tools available to create this data from WGS and RNA sequencing BAM files. Yet sometimes some of the data types could not readily be available for the samples under investigation. Necessity or optionality of each file type:

Expression files: This file is required and needs to be provided by the user. Revana assesses the gene expression to identify upregulated genes or regions (OHE: Outlier High Expression) and thus also regulatory events.

Marker files: This file is required and needs to be provided. Revana uses SNP markers to assess allele-specific expression (ASE), which is a criterion in its algorithm to identify

regulatory events. If no germline/control data is available, it might be sufficient to just provide the somatic single nucleotide variants as input.

SV / CNA / somatic SNV files: Each of these file types are technically optional. Yet, some mutation data needs to be provided to evaluate its regulatory potential and thus seek out regulatory variants. If any of these data types are not available, please provide an empty file with the headers as described in the documentation.

Copy number file: This input file is optional. Sometimes copy number variations may be available, while a complete genome-wide copy number file is not. Provide data for as many genomic regions as possible within this file to increase result accuracy. If no copy number data is available provide a blank file with the headers as described in the documentation.

The ChIP-Seq and GeneHancer files are optional and should only be provided if you want to use the particular functionality. See the respective sections on how to run Revana without these inputs.
The FIMO motif reference file and the FIMO Motif Id - TF Gene Name Conversion Table File are only required when using the transcription factor binding site analysis feature.
TAD files must be provided. Generally, TAD data is available online for many different tissue types (e.g. Hi-C boundary data from encode.org). Use a tissue type as closely related to the tumors under analysis as possible. Alternatively, human embryonic stem cell data can be used. While not encouraged, as a last resort 100 KB bins can be used to simulate TADs across the genome.

Reference files for the whole cohort

Gene Annotation Reference file

This file provides Revana with the required gene annotation. Besides the gene names types and genomic locations, it includes information about imprinting and cancer gene status. It can be created manually or by Revana from public downloads (see Create Required reference/input data with Revana). The file is formatted as tab separated values with header and contains the following columns:

Column name	Description	Example
chrom	chromosome of the gene in “UCSC style”	chr12
start	start of the gene	34890132
end	end of the gene	34895132
width	width of gene	5000
strand	strand of gene	+
gene_name	name of gene	PRDM6

gene_type	Gene type: Gene type of protein coding genes must be “protein_coding”	protein_coding
imprinting_status	Imprinting status of the gene: All genes with imprinting status other than “no_imprinting” are considered imprinted	imprinted
*imprinting_expressed_allele**	*allele that is expressed if gene is imprinted*	*Maternal*
*cancer_gene_role_in_cancer**	*Role of the gene in cancer development, if it is considered a cancer relevant gene*	*TSG*
is_cancer_gene	logical value (TRUE/FALSE) if the gene is a cancer relevant gene	TRUE

* These columns are annotative only. Thus, no calculations are based on their values.

Exon Annotation Reference file

This file provides Revana with the required exon annotation. It can be created manually or by Revana from public downloads (see Create Required reference/input data with Revana). The file is formatted as tab separated values with header and contains the following columns:

**Column name**	**Description**	**Example**
chrom	chromosome of the exon in “UCSC style”	chr12
start	start of the exon	34890132
end	end of the exon	34895132
gene_name	gene_name of the gene that the exon belongs to	PRDM6

FIMO Motif Reference File

This file is only required, if you plan on using the transcription factor binding site analysis feature, otherwise please skip the section.

FIMO requires a Motif file in .MEME format. Transcription factor motif files can be acquired from https://hocomoco11.autosome.org/ for different species in different formats. Download the appropriate file for your analysis, e.g. from https://hocomoco11.autosome.org/final_bundle/hocomoco11/core/HUMAN/mono/HOCOMOCOv11_core_HUMAN_mono_meme_format.meme .

FIMO Motif Id - TF Gene Name Conversion Table File

This file is only required, if you plan on using the transcription factor binding site analysis feature, otherwise please skip the section.

As FIMO motif IDs do not represent the gene names of the underlying transcription factors, Revana needs to be provided with a conversion table. Conversion tables can easily be created from annotation files available from download under https://hocomoco11.autosome.org/ e.g. https://hocomoco11.autosome.org/final_bundle/hocomoco11/core/HUMAN/mono/HOCOMOCOv11_core_annotation_HUMAN_mono.tsv . For subsequent conversion to the format required by Revana see Create Required reference/input data with Revana. The file is formatted as tab separated values with header and contains the following columns:

Column name	Description	Example
motif_id	the motif ID as used in the reference MEME file	AHR_HUMAN.H11MO.0.B
tf_gene_name	transcription factor gene name associated with the motif	AHR

GeneHancer Reference File

This file provides Revana with the GeneHancer dataset. It can be obtained via download from https://www.genecards.org/GeneHancer_Version_4-4 . Alternatively contact Simon Fishilevich from the GeneCards team to obtain the most current version of the data (email: simon.fishilevich@weizmann.ac.il). It is recommended to filter the provided dataset for double elite only entries. To format the reference files to the Revana required input (see Create Required reference/input data with Revana). The file is formatted as tab separated values with header and contains the following columns:

Column name	Description	Example
chrom	chromosome of the GeneHancer region in “UCSC style”	chr12

*feature_name**	*the name of the feature*	*Enhancer*
start	start of the GeneHancer region	12300000
end	end of the GeneHancer region	12301000
*score**	*score of the GeneHancer element*	*9.8*
genehancer_id	ID of the GeneHancer element	GH0000001
connected_gene	the gene regulated by this GeneHancer element	PRDM6
*connected_gene_score**	*the score of the gene to GeneHancer association*	*19.3*
*is_elite**	*is the GeneHancer element considered to be of elite evidence*	*TRUE*
*is_association_elite**	*is the GeneHancer element's association to the gene considered to be based on elite evidence*	*TRUE*

* These columns are annotative only. Thus, no calculations are based on their values.

If you want to run Revana without the GeneHancer functionality, set the *genehancer_ref_file_path* argument in the *run_analysis* function argument to *NULL*.

Create Required reference/input data with Revana

Marker file

In case that the RNA read counts for the marker SNPs are not readily available, Revana provides a function that extracts RNA read counts from the RNA-seq .bam file and adds them to the marker file.

```
add_RNA_read_count_to_markers(
# path to the RNA-seq .bam file
RNA_bam_file = RNA_bam_file,
```

```
# path to the marker file without the RNA read counts,
marker_file_without_RNA_read_count,
# output path for the new marker file with RNA read counts added
new_marker_file_path)
```

Extracting SNP marker read counts from large RNA seq files is a computational expensive operation. The function might therefore take some time to run. The marker file as input for this function must be formatted as described above, except without the columns “reads.RNA.ref” and “reads.RNA.alt”.

Gene/Exon Annotation Reference File

Revana helps create the required gene/exon annotation reference files. You will need to provide the following files. A Gencode GTF gene annotation file matching to your genome build version (in our case hg19) is required and can be downloaded from https://ftp.ebi.ac.uk/pub/databases/gencode/Gencode_human/release_40/GRCh37_mapping/gencode.v40lift37.annotation.gtf.gz . If you provide a different genome than hg19 build see Different genome build.
Additionally, Revana requires gene imprinting data available under https://www.geneimprint.com/site/genes-by-species.Homo+sapiens and cancer gene data from https://cancer.sanger.ac.uk/census . If you are not able to download these files, you can set the respective path arguments in the following function to an empty string (“”). This will allow you to create the required reference file without gene imprinting and/or cancer gene data, but is not recommended.

The following function creates the required gene and exon annotation reference files

```
prepare_gene_annotation_ref_file(
# path to the gencode GTF file
gencode_gtf_file_path = gencode_gtf_file_path,
# path to the imprint_genes_file_path OR empty string (“”)
imprint_genes_file_path = imprint_genes_file_path,
# path to the cancer gene file OR empty string (“”)
cancer_gene_file_path = cancer_gene_file_path,
# output path for the gene annotation file
ref_file_output_path = ref_file_output_path,
# output path for the exon annotation
ref_file_output_path_exons = ref_file_output_path_exons)
```

GeneHancer Reference File

To acquire the GeneHancer reference file, it is recommended to contact the GeneCard team via an online form under https://www.genecards.org/Guide/DatasetRequest or via email to Simon.Fishilevich@weizmann.ac.il . This is the only way to get the most recent version of the dataset as well as elite status annotation. We recommend filtering the GeneHancers by double elite status only.
Alternatively, a less current version of GeneHancer can be downloaded from https://www.genecards.org/GeneHancer_Version_4-4 .

The GeneHancer data can then be converted into the format as required by Revana via the following functions.

Import the data from Excel:

```
library("readxl")
genehancer_data <-
readxl::read_excel("path/to/Genehancer_version_X.xlsx")
```

Import the data from GFF format:

```
library("readr")
genehancer_data <- readr::read_tsv("path/to/genehancer.gff")
```

Supply paths for elite status data if available

```
genehancer_element_elite_status_file_path <-
"path/to/Genehancer_element_elite_status.txt"
genehancer_gene_associations_scores_file_path <-
"path/to/Genehancer_gene_associations_scores.txt"
```

Create GeneHancer Ref file for Revana:

```
prepare_genehancer_ref_file(
# import data like described above
genehancer_data,
# intended output path for the Genehancer Ref file for Revana
output_path,
# if elite status data is not avaible, set this argument to NULL
genehancer_element_elite_status_file_path =
genehancer_element_elite_status_file_path,
# if elite status data is not available, set this argument to NULL
genehancer_gene_associations_scores_file_path =
genehancer_gene_associations_scores_file_path,
# if elite status data IS available,
# consider only using double elite Genehancer elements for Revana by
# setting this argument to TRUE
keep_only_double_elites = FALSE,
# should the liftover from hg38 to hg 19 coordinates be skipped
skip_lifting_over = FALSE
)
```

GeneHancer coordinates are provided according to GRCh38 (hg38). By default, this function (*prepare_genehancer_ref_file*) lifts over the coordinates to GRCh37 (hg37). If you plan to run Revana with GRCh38 coordinates, set the *skip_lifting_over* argument in the function to TRUE.

FIMO Motif Id - TF Gene Name Conversion Table File

Revana helps create this file from annotation files available for download under https://hocomoco11.autosome.org/ e.g. https://hocomoco11.autosome.org/final_bundle/hocomoco11/core/HUMAN/mono/HOCOMOCOv11_core_annotation_HUMAN_mono.tsv . After the download convert the file to the required format with the following function:

```
prepare_motif_id_tf_gene_name_table_from_HOCOMOCO_annotation_tsv(
# path to the annotation file
annotation_file_path = annotation_file_path,
# output path for the conversion table reference file
output_path = output_path)
```

Run Revana

With all input and reference prepared as described above, Revana can be easily run within R.

First load the package into the R workspace.

```
library(revana)
```

Then run Revana with the following command:

```
run_analysis(
# path to the paths file
paths_file_path = paths_file_path,
# output directory to store results
output_dir = output_dir,
# path to the gene annotation file
gene_annotation_ref_file_path = gene_annotation_ref_file_path,
# path to the exon annotation file
gene_annotation_exons_ref_file_path =
gene_annotation_exons_ref_file_path,
# path to the TAD file
TAD_file_path = TAD_file_path,
# path to the FIMO motif ref file OR NULL
fimo_motif_ref_path = NULL,
# Path to the
# FIMO Motif ID - TF gene name conversion table file
# OR NULL
motif_id_tf_gene_name_table_path = NULL,
# path to the GeneHancer ref file path OR NULL,
genehancer_ref_file_path = genehancer_ref_file_path,
# path to the ChIP-Seq file OR NULL
chipseq_file_path = chipseq_file_path,
#name of the included subgroup or cohort e.g. medulloblastoma
subgroup_name = “MEDULLOBLASTOMA”
# whether TF binding site analysis is supposed to be run
run_tf_binding_site_analysis = FALSE,
# reference genome - provide NULL for hg 19
reference_genome = NULL
# should verbose logging be activated?
verbose = FALSE,
# should parallelization (multi-threading) be used to speed up
Revana?
use_parallelization = TRUE)
```

Revana can also be run without providing ChIP-Seq or GeneHancer files. In this case set the respective arguments to NULL.

After Revana has been run, the interactive HTML report can be generated like this:

```
create_results_HTML_report (
# output directory, where the HTML report should be stored
HTML_report_output_dir_path,
# results paths file - see below
output_paths_file_path = output_paths_file_path,
# whether TF binding site analysis has been conducted before
```

```
has_run_tf_binding_site_analysis = FALSE,
# should parallelization (multi-threading) be used to speed up Revana?
use_parallelization = FALSE)
```

The `output_paths_file_path` argument describes the path to the results paths file. After Revana has been run, it creates a file containing all the paths of the established results. To supply fewer arguments to the function, this file is designed to be used as input for the HTML report generation.
If several tumor subgroups are to supposed to be analysed within one HTML report, the results paths files have to be merged before supplying them as argument to the `create_results_HTML_report` function. This can be done like this

```
merge_results_paths_files(list_of_results_paths_file_paths=list(path_subgroup1, path_subgroup2), output_path_merged_results_file)
```

Different genome build

Revana uses the reference genome for transcription factor binding site analysis, visualizations and the IGV.js plugin. By default, Revana uses the genome assembly GRCh37 (hg19). If the user intends to use a different reference genome (e.g. because the supplied input date refers to a different assembly), make the following adaptions to the standard workflow.

- Use a compatible gene annotation from Gencode. For GRCh38 (hg38) you find the corresponding GTF file for download under https://ftp.ebi.ac.uk/pub/databases/gencode/Gencode_human/release_40/gencode.v40.annotation.gtf.gz
- Create the genehancer reference file in GRCh38 format by using

```
prepare_genehancer_ref_file(
...,
# should the liftover from hg38 to hg 19 coordinates be skipped
skip_lifting_over = TRUE
)
```

- Supply a reference_genome argument to the *run_analysis* function, e.g. for GRCh38 (hg38):

```
# install hg38 genome if not available yet
if (!require("BiocManager", quietly = TRUE))
    install.packages("BiocManager")

BiocManager::install("BSgenome.Hsapiens.UCSC.hg38")
reference_genome <- BSgenome.Hsapiens.UCSC.hg38::Hsapiens

run_analysis(
paths_file_path = paths_file_path,
gene_annotation_ref_file_path = gene_annotation_ref_file_path,
gene_annotation_exons_ref_file_path =
gene_annotation_exons_ref_file_path,
fimo_motif_ref_path = fimo_motif_ref_path,
TAD_file_path = TAD_file_path,
chipseq_file_path = chipseq_file_path,
genehancer_ref_file_path = genehancer_ref_file_path,
```

```
run_tf_binding_site_analysis = FALSE
subgroup_name = "SUBGROUP_NAME"
# GRCh38 genome assembly is used as follows
reference_genome = reference_genome
)
```

- Supply the genome name when using create_results_HTML_report. E.g. for GRCh38 (hg38):

```
create_results_HTML_report(
HTML_report_output_dir_path,
output_paths_file_path = output_paths_file_path,
has_run_tf_binding_site_analysis = FALSE,
genome_name = "hg38")
```

Transcription Factor (TF) Binding Site Analysis

Transcription factor binding site analysis can be a useful feature to understand the potentially underlying mechanisms in regulatory somatic SNV candidates. In the context of regulatory variant detection, this feature has previously been established in cis-X (Liu et al., 2020). Its ability to predict genuine regulatory variants from the pool of non-coding candidate SNVs is limited though.

ROC (matched via gene TADs)

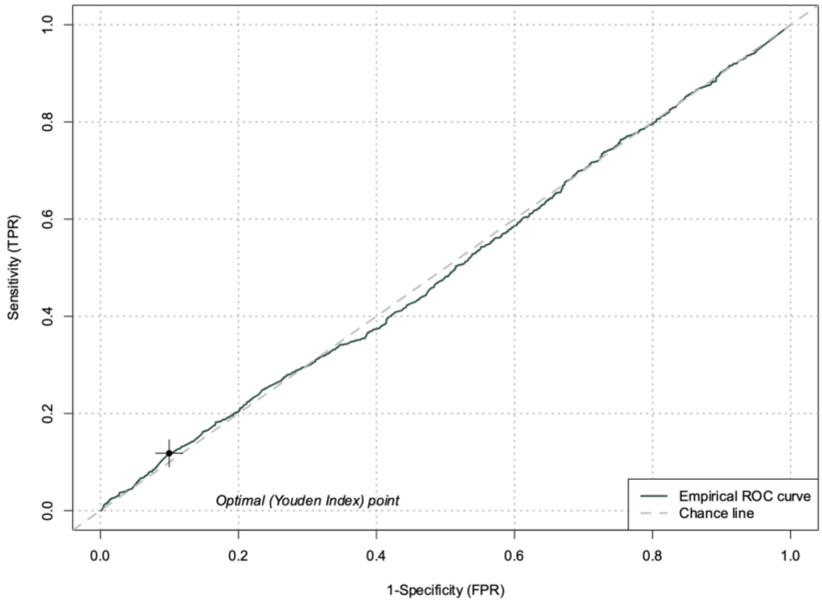

ROC (matched via genehancer)

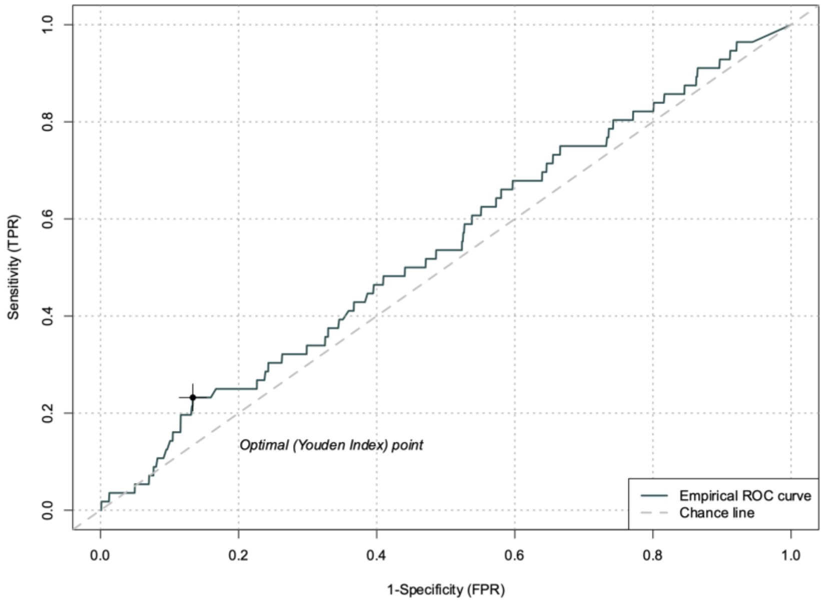

Receiver Operating Curves (ROC curves) of varying maximum TF binding score differences: FIMO provides a score of binding affinity for several transcription factors towards reference and alternate sequences of somatic SNVs. The maximum difference of alternate and reference affinity scores across all transcription factors and matched SNVs is calculated for each gene. The ROC shows that TF binding site can predict regulatory impact of SNVs only marginally better than a random predictor (dashed line).

Nevertheless, transcription factor binding site analysis can be a useful descriptive feature. Revana provides means of TF binding site analysis in a similar, but slightly enhanced fashion as cis-X. It uses the FIMO (Grant et al., 2011) tool from the MEME Suite to conduct the TF motif analysis.

Depending on the number of somatic SNVs of the tumors under investigation, TF binding analysis can be a computationally expensive operation and increase time, memory, and space consumption of Revana significantly. It is therefore disabled by default. To use the feature set *run_tf_binding_site_analysis = TRUE* to Revana's *run_analysis* function and *has_run_tf_binding_site_analysis = TRUE* to Revana's *create_results_HTML_report* function. **As the transcription factor binding site analysis feature uses the MEME suite as an external dependency, the user needs to have it installed and accessible in the current environment PATH.** You can find instructions on how to install MEME suite under https://meme-suite.org/ . Make sure to have the required reference files prepared ("FIMO Motif Id - TF Gene Name Conversion Table File" and "FIMO Motif Reference File").

Revana Workflow in Detail

Data input and preparation

Revana requires processed data from deep WGS and RNA-seq for a cohort of tumor samples as input. Tab-separated value files providing somatic and germline point mutation markers, copy numbers, structural variants, somatic SNVs and InDels and gene expression feature counts are used as input and need to be created prior to the application of Revana with any of the many

available tools (see Input formats). Input files should be matched to either of the reference genomes GRCh37 (hg19) or GRCh38 (hg38).
The demonstration data of 128 published medulloblastoma samples was aligned to human reference genome hg19 and the DKFZ in-house standardized analysis pipeline provided the required inputs. In this process, structural variant (SV) data was created with SOPHIA (Toprak, 2019), copy numbers and copy number alterations (CNAs) were called using ACESeq (Kleinheinz et al., 2017). Germline and somatic SNVs were called by an in-house algorithm based on mpileup and bcftools (Li et al., 2009). Indels were called by Platypus (Rimmer et al., 2014). Expression data was called using an in-house algorithm, with the tool featureCounts (Liao et al., 2014).
As the TAD reference file, we used data for human cerebellar astrocytes from ENCODE (The ENCODE Project Consortium, 2012) with the IDENTIFIER ENCFF306YQN.

Identification of cis-activated genes

Revana adopts the approach used by Liu et al., combining allele specific expression (ASE) and outlier high expression (OHE) to identify cis-activated candidate genes, that are potentially subject to variant-based upregulation.
First, SNP markers are filtered for sufficient read coverage ($n_{RNA\_reads} > 10$ and $n_{WGS\_reads} > 10$) and heterozygous presence in the genome ($0.3 \leq BAF \leq 0.7$). The allele frequencies of heterozygous SNP markers will then be used to calculate the p-values of ASE in comparison to a balanced expression model established by Liu et al.:

$$P(k, N) = B(k, N, P = 0.5) \times G\left(k, \mu = 0, \sigma = 10.8\left(1 - e^{-\frac{N}{105}}\right)\right)$$

Remarks:

B = Binomial Distribution
G = Gaussian Distribution
× = convolution operator
k = minor/major allele read count
N = RNA-seq read coverage (= total read count)

After summarizing the marker statistics on each gene and correcting for multiple testing via the Adaptive Benjamini-Hochberg procedure only genes with an FDR < 0.05 are considered allele-specifically expressed. If due to the structure of the data the adaptive Benjamini-Hochberg procedure fails, the more strict Bonferroni correction is used to ensure an FDR < 0.05.
Additionally, for genes that were annotated to be known cancer relevant genes, are considered to be allele-specifically expressed if the combined p-value of allele specific expression is less than 0.05 .
To raise the power of ASE detection, when insufficient SNP markers were available, a SNP marker run algorithm from Liu et al was reimplemented in R. This allows to use a series of monoallelically expressed SNP markers in the immediate vicinity of the gene to also determine ASE status.

For outlier high expression (OHE) Revana provides a procedure independent of precalculated gene expression reference matrices. It determines OHE using a Leave-One-Out test against a pre-filtered set of other tumor samples of the cohort:

$$t_i = \frac{x_i - \bar{x}_i}{\sqrt{s_i^2(1 + (n-2)^{-1})}}$$

Remarks

t_i = outlier t-statistic of sample i
x_i = expression of sample i
$\bar{x}_i$ = mean of expression of all remaining samples (besides sample i)
s_i = standard deviation of expression of all remaining samples (besides sample i)
n = total number of samples

Preferably a subset of biallelically expressed samples, cleaned from outliers, was used, if it was of sufficient size (default threshold: $n_{filtered\_reference\_samples} >= 10$). As this procedure was designed by Liu et al. to determine OHE, it was also implemented in Revana as the standard approach. In an alternative approach, that is unique to Revana, expression values are first corrected for copy number changes before undergoing the OHE test. First, exon regions of each gene are summarized into non-overlapping exon units. Genomic copy-numbers are then averaged within these regions to the determine the average copy number of the gene. The copy-number corrected expression values are subsequently calculated as the quotients of expression and average gene copy number. This approach helps to differentiate between genes upregulated by regulatory variants and genes with high expression due to copy number amplifications.

Finally, genes are considered cis-activated in their respective sample, when the following criteria is fulfilled. Besides having to be considered allele-specifically and outlier high expressed, generic genes must have an expression of more than 5 FPKM, while expression of cancer related genes must exceed 1 FPKM. To further assert allele-specific expression, the mean allelic imbalance is taken into account and has to be greater than 0.3 for diploid genes and 0.2 for genes within copy number altered regions. Genes that are annotated to be subject to genomic imprinting are excluded and never assigned to be cis-activated.

Associating genes with non-coding, regulatory variants

To identify potential regulatory somatic variants responsible for the upregulation of cis-activated genes, Revana matches genes and variants using three alternative approaches. As also utilized in cis-X, it first regards variants of the same Topologically Associating Domain (TAD). Here, cis-X prioritized SVs and CNAs and only considered somatic SNVs/InDels if no other variant type could be detected. Yet, the absence of larger variants alone does not render every neighboring SNV or InDel a feasible candidate, since somatic point mutations are frequent events in cancer genomes. Transcription Factor Binding Site Analysis with FIMO (Grant et al., 2011) may allow a more rigorous selection of SNVs, but does not detect regulatory variants significantly better and should therefore be regarded as descriptive (see Transcription Factor (TF) Binding Site Analysis).

Conversely, Revana associates genes irrespective of the gene’s cis-activation status with all types of variants from the same TAD including SVs, CNAs and somatic SNVs/InDels. This way, for each mutation type the potential for upregulation of neighboring genes can be estimated and compared.

Mutation Type	matched via gene TAD	matched via GeneHancer	matched via ChIP-Seq
any mutation	OddsRatio: 1.129 p-value: 2.350e-04	OddsRatio: 3.446 p-value: 5.847e-70	OddsRatio: 1.856 p-value: 4.487e-18
structural variant (SV)	OddsRatio: 2.226 p-value: 9.041e-14	OddsRatio: 3.863 p-value: 8.986e-30	OddsRatio: 2.678 p-value: 2.685e-19
copy number alteration (CNA)	OddsRatio: 2.016 p-value: 1.549e-17	OddsRatio: 4.259 p-value: 3.009e-65	OddsRatio: 1.928 p-value: 1.766e-19
somatic SNVs/InDels	OddsRatio: 1.108 p-value: 2.093e-3	OddsRatio: 2.938 p-value: 1.228-16	OddsRatio: 1.804 p-value: 4.475e-06
"relevant" somatic SNV/InDels*	Odds Ratio: 1.105 p-value: 3.611e-3	OddsRatio: 2.991 p-value: 9.013e-16	Odds Ratio: 1.747 p-value: 7.684e-05

*Relevant somatic SNVs/InDels in this context describe SNVs, that introduce new significant transcription factor binding sites to transcription factors with a minimum gene expression of > 5 FPKM in the same tumor sample.

A second approach exploits the GeneHancer dataset (Fishilevich et al., 2017), which provides gene specific regulatory regions. Promoter and enhancer elements of each gene under investigation allow for cross-TAD matching of SVs, CNAs and SNV/InDels as well as a more selective choice of point mutations within the same TAD. To be more precise, SVs and CNAs will also be matched to the gene if they share a TAD with gene-associated GeneHancer regions that is potentially distinct from the TAD of the gene. SNV/InDels will be matched by exact overlap of the regulatory region and thus are expected to be more likely to have regulatory influence on the target gene.

For the analysis of our medulloblastoma demonstration dataset we used the most recent version (5.4) of GeneHancer, provided by Simon Fishilevic et al from the GeneCards team, and filtered it for double-elite status GeneHancer elements only.

Although very useful, the GeneHancer dataset is not specific for any tissue or cancer entity. Any alternative tissue-specific or even sample-specific input providing gene-specific regulatory regions represents the ideal input dataset and could be alternatively used.

Even if gene associated regulatory regions are not available, in many cases regulatory active regions might have been experimentally identified for a tumor entity or subgroup. H3K27ac chromatin immunoprecipitation with DNA sequencing (ChIP-Seq) can provide this data appropriately to the cohort under study.
Integration of this ChIP-Seq data track reflects the third and optional approach for gene-to-variant matching. It is additionally used for result annotation and visualization.
For the investigated medulloblastoma cohort, previously published ChIP-Seq data is available (Lin et al., 2016) and has been integrated into the analysis.

Automatic report generation and data visualization

Subsequently, Revana creates a comprehensive HTML report that helps evaluate, prioritize, and visualize candidate events and allows to compare results of different tumor subgroups. Multiple figures summarize the composition of the cohort and facilitate quality control of the included samples.

Bar charts summarize the composition of the cohorts studied, the prevalence of different variant types per sample and subgroup, as well as expression profiles of the included samples. Principal component analysis (PCA) of gene expression allows to assess the comparability of RNA feature counts within the cohort and is useful to discover and subsequently address batch effect distortions.

JavaScript-powered interactive illustrations show recurrently cis-activated genes across the cohort, highlight samples with potentially regulatory variants of distinguishable kinds and provide multiple sorting and filtering options. We consider this the most useful approach to prioritize between the discovered genes and quickly elucidate genes recurrently affected by comparable regulatory mechanisms.
A summary of recurrent SV juxtapositions as in Rheinbay et al. provides additional evidence for important recurring regulatory candidate events.

SV TAD juxtaposition	N of samples	sample IDs	N of samples with cis-activated genes	affected cis-activated genes
chr8:126800001-127840000 <-> chr8:128800001-129600000	3	ICGC_MB198, ICGC_MB248, ICGC_MB50	2	ICGC_MB198: PVT1; ICGC_MB248: PVT1
chr8:127880001-128760000 <->	4	ICGC_MB198, ICGC_MB248,	2	ICGC_MB198: PVT1; ICGC_MB248: PVT1

chr8:128800001-129600000		ICGC_MB50, ICGC_MB94		
chr17:71400001-72880000 <-> chr17:77760001-79080000	3	ICGC_MB224, ICGC_MB243, ICGC_MB91	1	ICGC_MB243: TBC1D16
chr2:121040001-122600000 <-> chr2:123320001-124720000	2	ICGC_MB250, ICGC_MB50	1	ICGC_MB50: INHBB
chr2:121040001-122600000 <-> chr2:125400001-126400000	2	ICGC_MB243, ICGC_MB250	1	ICGC_MB243: CLASP1, CNTNAP5
chr2:122640001-123280000 <-> chr2:124760001-125360000	2	ICGC_MB243, ICGC_MB250	1	ICGC_MB243: CNTNAP5
chr2:222880001-223600000 <-> chr2:223640001-224760000	2	ICGC_MB106, ICGC_MB250	1	ICGC_MB250: ACSL3
chr5:86760001-88040000 <-> chr5:88080001-89000000	2	ICGC_MB19, ICGC_MB199	1	ICGC_MB199: MEF2C

For each discovered gene candidate a separate document shows the recurrence of somatic mutations, the expression and allelic imbalance of the gene across the investigated samples and tumor subgroups (if provided by the user) as well as more explicit statistics. A gene locus plot illustrates all present somatic variants and thus helps interpreting the genomic events. Finally, exploiting the powerful Integrative Genome Viewer (IGV) Plugin (Robinson et al., 2020), the gene TAD as well as other distant breakpoint loci can easily be investigated in an interactive way. Available alignment files can be loaded directly into the HTML report and greatly simplify common follow-up workflows like visual inspection in IGV.

Moreover, the report creation tool allows using custom R plugins. In case of the analysis of demonstration data this facilitated easy integration of Ace-Seq-generated tumor copy number profiles of the investigated samples.

To view the generated report of the demonstration dataset visit https://github.com/KiTZ-Heidelberg/revana-demo-report

Supplementary References

Grant, C.E., Bailey, T.L. and Noble, W.S. FIMO: scanning for occurrences of a given motif. *Bioinformatics* 2011;27(7):1017-1018.

Kleinheinz, K., *et al.* ACEseq – allele specific copy number estimation from whole genome sequencing. In.: Cold Spring Harbor Laboratory; 2017.

Li, H., *et al.* The Sequence Alignment/Map format and SAMtools. *Bioinformatics* 2009;25(16):2078-2079.

Liao, Y., Smyth, G.K. and Shi, W. featureCounts: an efficient general purpose program for assigning sequence reads to genomic features. *Bioinformatics* 2014;30(7):923-930.

Lin, C.Y., *et al.* Active medulloblastoma enhancers reveal subgroup-specific cellular origins. *Nature* 2016;530(7588):57-62.

Liu, Y., *et al.* Discovery of regulatory noncoding variants in individual cancer genomes by using cis-X. *Nature Genetics* 2020;52(8):811-818.

Rimmer, A., *et al.* Integrating mapping-, assembly- and haplotype-based approaches for calling variants in clinical sequencing applications. *Nature Genetics* 2014;46(8):912-918.

Robinson, J.T., *et al.* igv.js: an embeddable JavaScript implementation of the Integrative Genomics Viewer (IGV). In.: Cold Spring Harbor Laboratory; 2020.

The ENCODE Project Consortium. An integrated encyclopedia of DNA elements in the human genome. *Nature* 2012;489(7414):57-74.

Toprak, U. Doctoral dissertation; 2019. Integrative Analysis of Omics Datasets.
